# Are Women Deciding against Home Births in Low and Middle Income Countries?

**DOI:** 10.1371/journal.pone.0065527

**Published:** 2013-06-14

**Authors:** Fiifi Amoako Johnson, Sabu S. Padmadas, Zoë Matthews

**Affiliations:** Centre for Global Health, Population, Poverty and Policy and Division of Social Statistics and Demography, University of Southampton, Southampton, United Kingdom; Aga Khan University, Pakistan

## Abstract

**Background:**

Although there is evidence to tracking progress towards facility births within the UN Millennium Development Goals framework, we do not know whether women are deciding against home birth over their reproductive lives. Using Demographic and Health Surveys (DHS) data from 44 countries, this study aims to investigate the patterns and shifts in childbirth locations and to determine whether these shifts are in favour of home or health settings.

**Methods and Findings:**

The analyses considered 108,777 women who had at least two births in the five years preceding the most recent DHS over the period 2000–2010. The vast majority of women opted for the same place of childbirth for their successive births. However, about 14% did switch their place and not all these decisions favoured health facility over home setting. In 24 of the 44 countries analysed, a higher proportion of women switched from a health facility to home. Multilevel regression analyses show significantly higher odds of switching from home to a facility for high parity women, those with frequent antenatal visits and more wealth. However, in countries with high infant mortality rates, low parity women had an increased probability of switching from home to a health facility.

**Conclusions:**

There is clear evidence that women do change their childbirth locations over successive births in low and middle income countries. After two decades of efforts to improve maternal health, it might be expected that a higher proportion of women will be deciding against home births in favour of facility births. The results from this analysis show that is not the case.

## Introduction

Do women in low and middle income countries use health facilities for childbirth consistently across their reproductive life? This question has not been examined systematically in resource poor settings where the levels of maternal mortality continue to remain high. The aim of this paper is to investigate the patterns and shifts in childbirth locations in low and middle income countries and to determine whether these shifts are in favour of home or health settings. This research is conceptualised within the safe motherhood initiative programme and targets 5a and 5b of the Millennium Development Goals (MDG) framework, which aim to increase skilled care at birth in hygienic and conducive environment with essential obstetric facilities as one of the strategies to reduce the levels of maternal mortality in the developing world [1]–[3]. There is evidence of increased uptake of institutional delivery care in low and middle income countries [4], although in most countries, this increase is not large enough to reach the MDG targets [2]. However, there is no systematic analysis on whether women continue to use health facilities for subsequent births. This may partly explain why the increase in institutional delivery care has been slow in some countries.

Every year, an estimated 358,000 women die from complications due to childbirth [1] – 99% of these occur in developing countries mostly at the time of birth [5]–[7]. Most of these deaths can be avoided if women had access to emergency obstetric care under the supervision of skilled health professionals [7]–[8]. Despite two decades of maternal health initiatives in the developing world, a high proportion of births continue to occur at home in unhygienic conditions without any skilled care and without the essential infrastructure needed to refer in the case of complications [9]–[11].

Poverty is one of the fundamental factors that explain high rates of home births in Africa and Asia. Women from poor households and marginalised communities lack access to proper maternity care and in settings where services are available tend to be constrained by high economic costs and poor quality of care. The decision against a facility birth is also influenced by household decision making and convenience, irrespective of the wealth factor [12]–[15]. Community perceptions and positive experiences associated with traditional birth attendants might also favour women to choose a home birth [16], although there is evidence that presence of even a trained traditional birth attendant is of little help to women who develop complications at birth [17]–[18]. On the other hand, there is inequality and inequity in facility births especially between women who give a birth in a government or public facility and private-for-profit institutions [14]. Any interventions focused on shifting skilled home-based to facility-based care should therefore consider a range of factors including social and cultural settings, equity, economic costs, acceptability, effectiveness and implications for health-care equity in both approaches [19].

We hypothesise that women who had a birth at home are unlikely to switch to a health facility for their subsequent birth and vice versa. If there is evidence that women are moving away from facilities in favour of home births, then this suggests that economic burden or poor quality of maternity care is driving women back to their homes for childbirth. On the other hand, if women are deciding against home for their subsequent birth, this might suggest experience of complications in their previous or current pregnancy or confidence in the healthcare system.

We analysed retrospective pregnancy histories from the Demographic and Health Surveys (DHS) conducted in 44 countries from sub-Saharan Africa, North Africa, Central, South and Southeast Asia, Latin America and the Caribbean to investigate the patterns and shifts in childbirth locations and to determine whether these shifts are in favour of home or health settings.

## Methods

### Data and study design

Data from the most recent DHS surveys conducted in 44 countries between 2000 and 2010 were selected for the analyses. Information on place of childbirth was available for all children born in the five years preceding the survey. To compare the patterns and shifts in childbirth locations, we selected women who had at least *two* births. Most women (over 80%) had two births within the five years preceding the survey. A total of 108,777 women from 29 countries in sub-Saharan Africa (representing 64% of the sample), 8 countries in South and Southeast Asia (26%), 4 countries in Latin America and Caribbean (6%) and 3 countries from North Africa and Central Asia (4%) were included in the analysis.

Two outcome measures were investigated (i) shifts in childbirth locations for successive births indicating movement from one place of birth to another (n = 108,777) and (ii) the direction of the shift, either home to facility or facility to home, conditional on women who changed their childbirth location (n = 15,006).

The analyses focus on three primary factors associated with switching behaviour; birth experiences (measured by parity), access to and the extent of maternity care services use (measured by proxy variables: frequency of antenatal visits and geographical location of maternal residence) and affordability of maternity services (household wealth as proxy variable) [7], [9], [10], [14], [20]. To measure household wealth, we used the standard DHS wealth quintiles based on asset ownership using principal component analysis [21]. Other primary factors such as quality of care and physical distance to health care services have not been considered due to lack of data availability [20].

Other relevant confounders were selected based on the existing literature that reported the determinants of maternal health care use in developing countries [7], [22]–[24]. These include maternal age, marital status, years lived in the current residence as a proxy variable to capture possible effects of recent migration, women's education, partners' education and geographical region of residence (sub-Saharan Africa, South and Southeast Asia, Latin America and Caribbean and Central Asia and North Africa).

In addition, we examined contextual factors related to both utilisation and provision of maternity care services [7]. These include indicators at country level to reflect women's autonomy and social status (percentage of females participating in the labour market; adult female mean years of schooling and total fertility rate); governments' commitment to health care (public expenditure on health as a % of GDP); the quality and availability of services within health systems (human resource density for health expressed as the number of physicians per 10000 population, percentage of births attended by skilled health personnel, infant mortality rate); and aggregate wealth (Gross National Income (GNI) per capita PPP US$) at the country level. We also examined the impact of financial pressure on governments (debt service measured as a % of GNI). External debt constrains the ability of many low and middle income countries to meet basic services including maternity care [25]. Over the past two decades, external debt in less developed countries has aggravated and often unmanageable, despite regular servicing which is done at the expense of key services including health, education, water, sanitation and food [26], [27].

The contextual data covering the period between 2000 and 2008 were collated from the Human Development Reports published by the United Nations Development Programme. To account for the time lag, all surveys were linked to the data closer to the point of observation.

### Statistical analysis

At the first stage of the analysis, odds ratios and their 95% confidence intervals, adjusting for clustering effects, were estimated to determine the odds of switching childbirth location versus not switching and the direction of switching (from home to a health facility and vice versa) for those who did switch the location. The complex sampling design of the DHS is accounted for, using the CSPLAN option in IBM SPSS Statistics software version 20 which controls for potential clustering effects in the bivariate analysis [28]. The second stage considered analysis of mothers who switched their childbirth place disaggregated by primary factors: parity (women with three or more children versus two children); number of antenatal visits (4 or more visits versus less than 4 visits including), household wealth status (bottom 40% versus top 60%) and geographical location of residence (rural versus urban). The final stage of the analysis considered a two level random intercept logistic regression to model the variations in switching from home to a health facility, adjusting for selected confounders and contextual factors.

Random intercept models [29] were fitted with women (level 1) nested within countries (level 2) to capture the potential unobserved heterogeneity at the country level. The regression considered a sequential approach to model building to understand how much of the variation in the direction of switching is explained by the primary factors, control and contextual variables. Information on the place of childbirth self-reported by mothers was believed to be fairly accurate since there is no reason to believe that a mother would misreport her place of childbirth particularly recent births.

## Results

**Figure 1:**
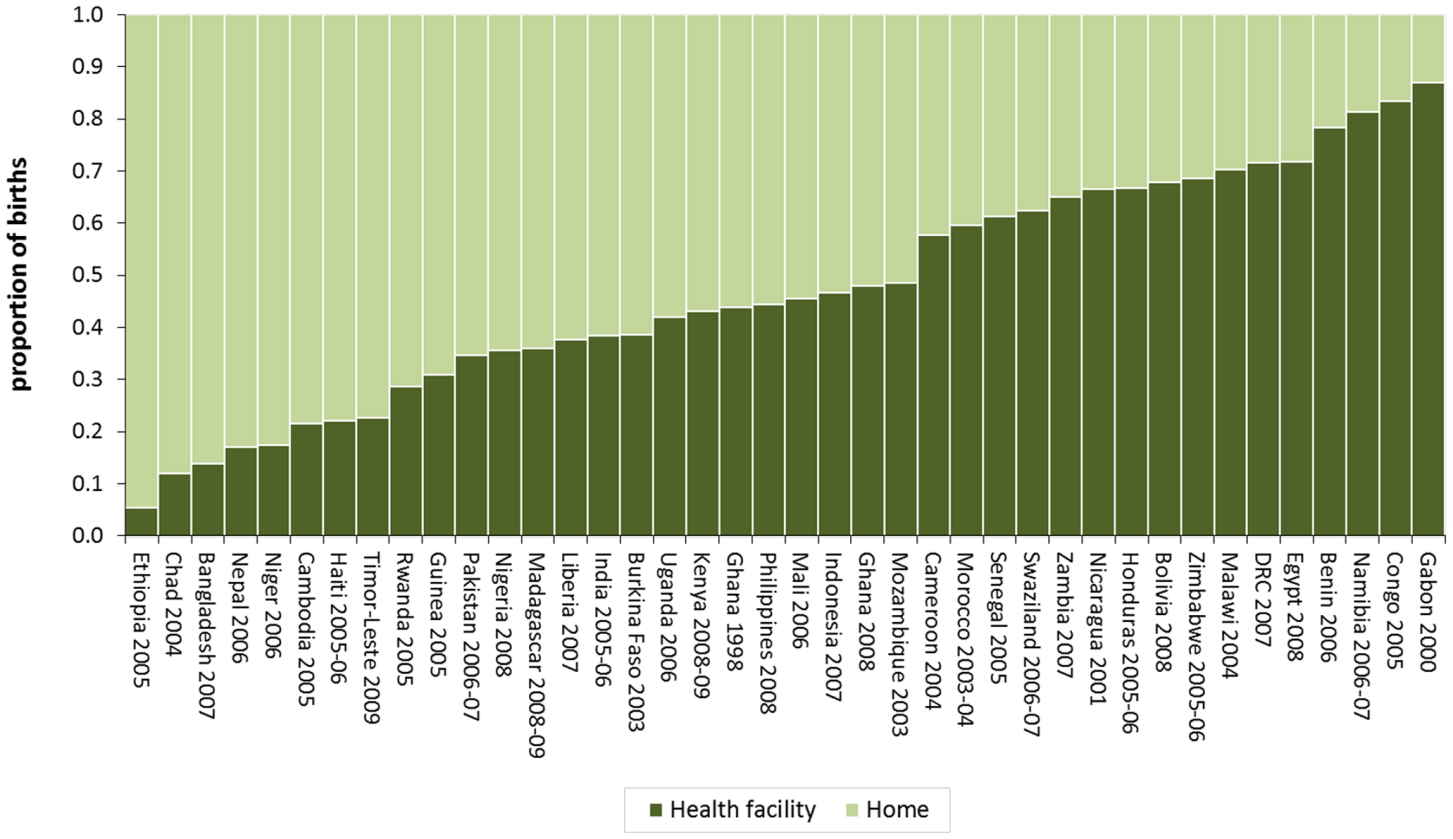
Place of childbirth for all births in the five years preceding the survey.

Institutional births vary widely across low and middle income countries (Figure 1). For example, in Namibia, Congo and Gabon facility births account for more than 80% of all births, while in Ethiopia, Chad and Niger they account for less than 20% of all births. Institutional births are generally uncommon in Asia. They vary from as low as 14% in Bangladesh to 47% in Indonesia (Figure 1).

**Figure 2:**
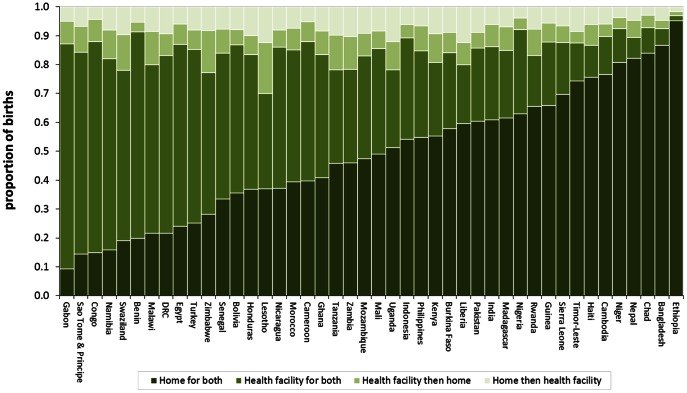
Choice of childbirth place for the last two successive births in the five years to the survey.

Women's choice of childbirth place is graphically illustrated in Figure 2. There is evidence of women changing their place of childbirth between home and health facility over successive births. In countries where overall levels of institutional births are low, switching place of childbirth is also low (Figure 2). This clearly indicates that women in these countries are not opting for alternatives choices instead continue giving birth at home. About 14% of women in low and middle income countries did switch their place of childbirth. In 38 countries, more than one-tenth of mothers switch their place of childbirth. This is more than one-fifth in Liberia, Malawi, Zambia, Tanzania, Uganda, Swaziland, Zimbabwe and Lesotho. At the regional level, 12.9% of mothers in South and Southeast Asia switch their place of childbirth, 13.9% in sub-Saharan Africa, 14.3% in Latin America and Caribbean and 13.8% in North Africa.

In 24 countries, a higher percentage of mothers switch from a facility to home (Table 1). In sub-Saharan Africa and North Africa, the shift is in favour of home against facility whereas in the Latin American and Caribbean region the direction of switch is towards facility. The patterns in South and Southeast Asia are evenly distributed. There are a number of countries where the movement is towards facility births, for example Ethiopia, Ghana, Sierra Leone, Indonesia, Bangladesh and Pakistan. However, none of these aggregate movements are large enough to be significant. Estimates from the pooled data show a balanced movement away from facility to home births and from home to facility births (7% each).

**Table 1:** Pattern of switching in the place of childbirth.

Country (year of survey)	Switched place of childbirth		Switch between home and health facility				Absolute difference [95% CI]H→F vs. F→H	Number of women
	No	Yes	H→H	F→F	F→H	H→F		
Bangladesh (2007)	92.5	7.5	86.7	5.8	3.0	4.6	1.6 [−6.9, 10.2]	1103
Benin (2006)	91.3	8.7	19.9	71.4	3.5	5.2	1.7 [−2.3, 5.8]	4789
Bolivia (2008)	86.9	13.1	35.6	51.3	5.3	7.8	2.5 [−3.8, 8.9]	1916
Burkina Faso (2003)	84.1	15.9	57.8	26.3	7.0	8.9	1.9 [−3.0, 6.8]	3024
Cambodia (2005)	89.7	10.3	76.6	13.1	4.3	6.0	1.7 [−4.6, 8.0]	1936
Cameroon (2004)	88.0	12.0	39.8	48.2	6.9	5.1	−1.8 [−7.5, 3.8]	2363
Chad (2004)	92.9	7.1	84.0	8.9	4.2	2.9	−1.3 [−7.9, 5.3]	1826
Congo (2005)	88.1	11.9	14.9	73.2	7.7	4.3	−3.4 [−12.1, 5.3]	1121
DRC (2007)	83.1	16.9	21.7	61.4	7.5	9.3	1.8 [−3.3, 6.9]	2762
Egypt (2008)	87.0	13.0	24.0	63.0	7.1	5.9	−1.2 [−6.5, 4.2]	2557
Ethiopia (2005)	97.0	3.0	95.2	1.8	1.4	1.6	0.1 [−5.1, 5.3]	2804
Gabon (2000)	87.2	12.8	9.3	77.9	7.9	4.9	−3.0 [−11.2, 5.2]	1180
Ghana (2008)	83.5	16.5	40.8	42.7	8.2	8.3	0.1 [−9.6, 9.9]	736
Guinea (2005)	87.8	12.2	65.8	22.0	6.7	5.5	−1.1 [−7.7, 5.5]	1687
Haiti (2005/06)	86.8	13.2	75.7	11.1	7.1	6.1	−1 [−7.9, 5.8]	1543
Honduras (2005/06)	83.5	16.5	36.9	46.6	6.6	9.9	3.4 [−2.3, 9]	2410
India (2005/06)	86.2	13.8	61.0	25.2	7.8	6.1	−1.7 [−4.1, 0.7]	12739
Indonesia (2007)	89.3	10.7	54.1	35.1	4.7	6.0	1.3 [−3.7, 6.4]	2925
Kenya (2008/09)	80.7	19.3	55.3	25.4	9.9	9.3	−0.6 [−7.1, 5.8]	1661
Lesotho (2009)	70.1	29.9	37.0	33.1	17.6	12.3	−5.2 [−14.8, 4.3]	754
Liberia (2007)	80.0	20.0	59.5	20.5	7.7	12.3	4.7 [−2.6, 11.9]	1455
Madagascar (2008/09)	85.0	15.0	61.6	23.4	8.2	6.8	−1.3 [−6.0, 3.4]	3273
Malawi (2004)	79.9	20.1	21.6	58.3	11.6	8.5	−3.1 [−7.9, 1.7]	3154
Mali (2006)	85.6	14.4	49.1	36.5	6.1	8.4	2.3 [−1.8, 6.5]	4444
Morocco (2003/04)	85.2	14.8	39.5	45.7	7.4	7.4	0.0 [−7.6, 7.6]	1242
Mozambique (2003)	83.1	16.9	47.4	35.7	7.8	9.1	1.4 [−3.6, 6.4]	2872
Namibia (2006/07)	82.1	17.9	16.0	66.1	9.9	8.0	−1.9 [−10.4, 6.5]	1012
Nepal (2006)	89.5	10.5	82.2	7.3	5.9	4.6	−1.2 [−8.5, 6.1]	1405
Nicaragua (2001)	86.1	13.9	37.1	49.0	5.8	8.1	2.3 [−4.5, 9.1]	1629
Niger (2006)	92.5	7.5	80.8	11.7	3.8	3.7	−0.2 [−5.2, 4.9]	2882
Nigeria (2008)	92.2	7.8	63.0	29.1	4.0	3.9	−0.1 [−3.0, 2.8]	8776
Pakistan (2006/07)	85.8	14.2	60.4	25.4	5.4	8.8	3.5 [−1.9, 8.9]	2750
Philippines (2008)	84.8	15.2	54.8	29.9	8.7	6.6	−2.1 [−8.9, 4.8]	1560
Rwanda (2005)	83.2	16.8	65.6	17.6	9.1	7.7	−1.3 [−6.5, 3.8]	2714
Sao Tome & Principe (2009)	84.3	15.7	14.4	69.9	9.0	6.7	−2.3 [−15.5, 10.8]	427
Senegal (2005)	83.9	16.1	33.5	50.4	8.3	7.7	−0.6 [−5.3, 4.1]	3259
Sierra Leone (2008)	87.6	12.4	69.7	17.9	5.9	6.5	0.6 [−6.6, 7.9]	1374
Swaziland (2006/07)	78.1	21.9	19.1	59.0	12.4	9.5	−2.9 [−13.6, 7.9]	607
Tanzania (2010)	78.2	21.8	45.8	32.4	12.0	9.8	−2.2 [−7.8, 3.4]	2224
Timor-Leste (2009)	87.5	12.5	74.4	13.1	4.0	8.5	4.4 [−1.0, 9.9]	3141
Turkey (2003)	85.3	14.7	25.2	60.1	6.8	7.9	1.1 [−7.5, 9.7]	966
Uganda (2006)	78.2	21.8	51.2	27.0	9.9	11.9	2.1 [−2.9, 7.1]	2772
Zambia (2007)	78.4	21.6	46.0	32.4	11.3	10.3	−1.0 [−6.9, 5.0]	1952
Zimbabwe (2005/06)	77.3	22.7	28.3	49.0	14.5	8.2	−6.3 [−14.9, 2.4]	1051
South and Southeast Asia	87.1	12.9	64.5	22.6	6.5	6.5	0.0 [−1.6, 1.7]	27559
sub-Saharan Africa	86.1	13.9	50.3	35.9	7.1	6.8	−0.3 [−1.3, 0.7]	68955
Latin America & Caribbean	85.7	14.3	44.5	41.2	6.2	8.1	2.0 [−1.2, 5.1]	7498
North Africa & Central Asia	86.2	13.8	28.4	57.8	7.1	6.7	−0.5 [−4.3, 3.4]	4765
All	86.3	13.7	52.7	33.7	6.9	6.8	−0.1 [−0.9, 0.7]	108777

H – Home, F – Health Facility.

The odds ratios and their corresponding 95% Confidence Intervals (CIs) for the direction of switch in place of childbirth by parity, antenatal visits, household wealth and residence are illustrated in Table 2. The results are conditional on women who switched their childbirth place, adjusting for clustering effects. In most countries, the odds of switching for high parity women are significantly in favour of a health facility than home when compared to women of low parity. Women who had four or more antenatal visits are more likely to switch from home to a health facility for their successive birth – statistically significant in 13 countries. The direction of switch did not vary significantly by wealth status, except in Haiti and Timor-Leste where switching is towards a health facility and in Liberia and Philippines the direction is towards home birth for the poorest 40%. Rural-urban differences are statistically significant only in 5 countries. In Liberia, Swaziland and Zimbabwe, rural mothers are less likely to switch from home to a health facility whereas in Namibia and Timor-Leste the direction is in favour of facility over home births for mothers living in rural areas.

**Table 2:** Odds ratios [95% CIs] for switching from home to a health facility adjusted for clustering.

Country	Parity (high vs. low parity)	Antenatal visits (4+ vs. no visits)	Wealth (bottom 40% vs. top 60%)	Residence (rural vs. urban)
Bangladesh	10.47 [3.14, 34.97]**	3.71 [1.22, 11.34] *	0.79 [0.31, 2.03]	1.00 [0.34, 2.94]
Benin	1.85 [1.1, 3.13]*	1.95 [1.28, 2.98] **	0.91 [0.60, 1.37]	1.52 [0.95, 2.41]
Bolivia	2.00 [1.17, 3.42]*	1.69 [1.00, 2.85] *	0.56 [0.31, 1.01]	0.84 [0.49, 1.44]
Burkina Faso	2.62 [1.61, 4.24]**	0.99 [0.60, 1.64]	0.91 [0.61, 1.35]	0.53 [0.25, 1.13]
Cambodia	4.18 [2.21, 7.92]**	1.45 [0.74, 2.84]	1.19 [0.64, 2.19]	0.93 [0.36, 2.39]
Cameroon	2.27 [1.29, 3.99]**	0.72 [0.45, 1.16]	0.89 [0.55, 1.42]	0.98 [0.60, 1.59]
Chad	1.70 [0.79, 3.67]	2.25 [1.12, 4.53] *	1.13 [0.50, 2.56]	0.59 [0.30, 1.16]
Congo	0.92 [0.44, 1.91]	2.49 [1.21, 5.11] *	1.05 [0.50, 2.22]	0.63 [0.27, 1.48]
DRC	1.41 [0.91, 2.21]	1.61 [1.10, 2.34] *	0.82 [0.56, 1.19]	1.42 [0.89, 2.25]
Egypt	3.19 [2.00, 5.08]**	0.77 [0.50, 1.21]	1.16 [0.74, 1.82]	1.06 [0.63, 1.78]
Ethiopia	4.29 [1.81, 10.14]**	0.76 [0.33, 1.77]	1.19 [0.43, 3.32]	1.83 [0.68, 4.97]
Gabon	1.33 [0.58, 3.05]	1.82 [0.90, 3.70]	0.71 [0.35, 1.42]	0.66 [0.32, 1.32]
Ghana	1.47 [0.66, 3.28]	1.68 [0.65, 4.31]	0.70 [0.33, 1.51]	0.94 [0.39, 2.28]
Guinea	3.62 [1.68, 7.80]**	1.64 [0.93, 2.90]	0.79 [0.44, 1.41]	0.78 [0.41, 1.48]
Haiti	6.66 [3.29, 13.5]**	1.06 [0.59, 1.91]	1.97 [1.06, 3.65] *	1.74 [0.97, 3.12]
Honduras	4.85 [2.87, 8.20]**	2.14 [1.34, 3.43] **	1.25 [0.79, 1.96]	0.86 [0.53, 1.40]
India	3.28 [2.73, 3.94]**	1.64 [1.35, 1.99] **	0.84 [0.70, 1.00]	0.69 [0.56, 0.84]
Indonesia	1.60 [0.95, 2.71]	1.52 [0.84, 2.77]	0.77 [0.46, 1.30]	0.91 [0.53, 1.58]
Kenya	3.24 [1.93, 5.44]**	1.39 [0.88, 2.19]	1.31 [0.83, 2.05]	1.29 [0.63, 2.62]
Lesotho	1.99 [1.12, 3.55]*	1.14 [0.64, 2.05]	0.94 [0.52, 1.67]	0.60 [0.24, 1.50]
Liberia	1.97 [1.13, 3.45]*	0.67 [0.30, 1.47]	0.43 [0.26, 0.71] **	0.52 [0.30, 0.90]*
Madagascar	1.57 [1.06, 2.33]*	1.91 [1.33, 2.72] **	1.01 [0.71, 1.44]	1.04 [0.60, 1.80]
Malawi	1.35 [0.95, 1.93]	1.29 [0.93, 1.78]	0.79 [0.57, 1.08]	0.51 [0.26, 1.00]
Mali	1.89 [1.31, 2.74]**	1.09 [0.79, 1.52]	0.97 [0.71, 1.32]	0.85 [0.58, 1.26]
Morocco	5.85 [2.95, 11.59]**	1.07 [0.51, 2.25]	0.67 [0.35, 1.26]	0.91 [0.48, 1.73]
Mozambique	1.41 [0.96, 2.07]	2.03 [1.41, 2.90] **	1.02 [0.72, 1.44]	1.20 [0.78, 1.84]
Namibia	1.69 [0.91, 3.14]	1.21 [0.62, 2.39]	1.70 [0.89, 3.25]	2.77 [1.04, 7.37]*
Nepal	3.99 [1.96, 8.11]**	1.33 [0.68, 2.61]	0.76 [0.37, 1.56]	1.36 [0.57, 3.26]
Nicaragua	3.36 [1.77, 6.36]**	1.75 [0.99, 3.11]	1.15 [0.64, 2.09]	0.95 [0.52, 1.74]
Niger	1.87 [1.00, 3.49]*	1.63 [0.85, 3.12]	0.66 [0.38, 1.15]	1.43 [0.81, 2.51]
Nigeria	1.91 [1.34, 2.73]**	1.56 [1.11, 2.18] **	1.14 [0.82, 1.57]	0.83 [0.60, 1.14]
Pakistan	3.56 [2.25, 5.63]**	0.96 [0.61, 1.50]	1.07 [0.71, 1.63]	0.90 [0.58, 1.40]
Philippines	3.29 [1.82, 5.95]**	0.81 [0.45, 1.45]	0.55 [0.32, 0.94] *	0.85 [0.50, 1.44]
Rwanda	5.73 [3.60, 9.11]**	1.39 [0.83, 2.32]	1.43 [0.97, 2.11]	1.32 [0.79, 2.20]
Sao Tome & Principe	12.71 [2.60, 62.15]**	0.38 [0.12, 1.17]	2.31 [0.82, 6.51]	2.67 [0.92, 7.76]
Senegal	3.58 [2.31, 5.55]**	0.93 [0.65, 1.33]	1.18 [0.82, 1.68]	1.39 [0.90, 2.15]
Sierra Leone	2.63 [1.32, 5.24]**	1.57 [0.73, 3.36]	1.02 [0.56, 1.87]	0.89 [0.47, 1.69]
Swaziland	2.79 [1.34, 5.81]**	1.33 [0.63, 2.81]	0.59 [0.30, 1.19]	0.33 [0.11, 0.93]*
Tanzania	1.85 [1.20, 2.84]**	1.11 [0.76, 1.62]	0.82 [0.57, 1.17]	1.80 [0.99, 3.25]
Timor-Leste	1.54 [0.96, 2.45]	1.40 [0.91, 2.14]	2.21 [1.27, 3.86] **	2.53 [1.64, 3.90]**
Turkey	5.59 [2.41, 12.96]**	2.10 [0.88, 5.00]	0.54 [0.23, 1.28]	0.74 [0.36, 1.53]
Uganda	1.81 [1.21, 2.71]**	1.63 [1.18, 2.25] **	–	0.80 [0.40, 1.60]
Zambia	1.72 [1.09, 2.72]*	2.64 [1.77, 3.92] **	0.89 [0.61, 1.30]	1.01 [0.63, 1.63]
Zimbabwe	1.30 [0.74, 2.29]	1.76 [1.01, 3.08] *	0.73 [0.41, 1.29]	0.35 [0.14, 0.87]*

** p<0.01,

* p<0.05;

reference category (switching from health facility to home).

Country-specific odds of switching from home to a health facility adjusting for primary factors, maternal age and education, partner's education and clustering effects are shown in Table 3. The results show that, in most countries, high parity and frequent antenatal visits are significantly associated with switching from home to a health facility. The effect of wealth is trivial, except in Bolivia, India and Philippines where the poor are less likely to switch from home to a health facility but more likely to switch from home to a health facility in Timor-Leste. The effect of residence is significant only in Niger, Timor-Leste and Zimbabwe. It has to be noted that even after adjusting for primary factors and other background characteristics, parity and antenatal care remain the key factors determining switching from home to a health facility.

**Table 3:** Odds ratios [95% CIs] for switching from home to a health facility adjusted for the primary factors, maternal age and education, partner's educational status and clustering.

Country	Parity	Antenatal visits	Wealth	Residence
	(high vs. low parity)	(4+ vs. no visits)	(bottom 40% vs. top 60%)	(rural vs. urban)
Bangladesh	6.45 [1.21, 34.50] *	6.28 [1.47, 26.75] *	0.76 [0.26, 2.22]	1.13 [0.40, 3.22]
Benin	2.21 [1.27, 3.85] **	4.24 [2.28, 7.87] **	0.87 [0.57, 1.34]	1.48 [0.93, 2.32]
Bolivia	3.11 [1.68, 5.77] **	2.64 [1.13, 6.16] *	0.38 [0.16, 0.91] *	1.45 [0.67, 3.08]
Burkina Faso	2.75 [1.63, 4.64] **	3.85 [1.64, 9.06] **	1.06 [0.68, 1.65]	0.50 [0.25, 1.01]
Cambodia	6.88 [3.03, 15.62] **	2.55 [0.93, 7.04]	1.13 [0.55, 2.30]	1.03 [0.44, 2.39]
Cameroon	1.81 [1.01, 3.26] *	1.02 [0.48, 2.13]	0.91 [0.54, 1.55]	1.02 [0.58, 1.77]
Chad	2.21 [1.04, 4.70] *	2.26 [0.90, 5.73]	1.70 [0.51, 5.61]	0.53 [0.21, 1.31]
Congo	1.24 [0.50, 3.04]	3.67 [1.51, 8.94] **	0.99 [0.36, 2.70]	0.75 [0.27, 2.06]
DRC	1.21 [0.73, 2.01]	1.74 [0.97, 3.11]	0.80 [0.54, 1.26]	1.63 [0.94, 2.82]
Egypt	3.81 [2.17, 6.67] **	0.86 [0.52, 1.43]	0.85 [0.50, 1.45]	0.97 [0.54, 1.75]
Ethiopia	3.02 [1.19, 7.72] *	2.73 [0.97, 7.70]	1.31 [0.42, 4.09]	1.05 [0.42, 2.59]
Gabon	0.86 [0.39, 1.92]	4.39 [1.16, 16.69] *	0.77 [0.33, 1.81]	1.14 [0.51, 2.58]
Ghana	2.04 [0.79, 5.26]	1.72 [0.61, 4.37]	0.62 [0.24, 1.57]	1.18 [0.40, 3.51]
Guinea	3.53 [1.58, 7.87] **	2.64 [0.91, 7.70]	1.20 [0.61, 2.34]	1.23 [0.57, 2.56]
Haiti	4.99 [2.16, 11.53] **	2.33 [0.65, 8.49]	1.95 [0.82, 4.62]	0.83 [0.37, 1.86]
Honduras	4.77 [2.81, 8.10] **	5.07 [2.50, 10.29] **	0.86 [0.45, 1.63]	0.73 [0.37, 1.43]
India	4.73 [3.65, 6.11] **	1.82 [1.32, 2.49] **	0.78 [0.61, 0.99] *	0.88 [0.71, 1.09]
Indonesia	1.82 [1.03, 3.21] *	1.28 [0.46, 3.58]	0.74 [0.42, 1.32]	0.85 [0.48, 1.51]
Kenya	3.14 [1.78, 5.53] **	3.13 [0.77, 12.71]	1.46 [0.86, 2.48]	0.69 [0.34, 1.39]
Lesotho	2.47 [1.27, 4.79] **	2.71 [0.97, 7.56]	1.35 [0.72, 2.51]	0.47 [0.17, 1.34]
Liberia	2.20 [1.13, 4.28] *	2.07 [1.03, 4.13] *	0.91 [0.51, 1.63]	0.59 [0.36, 1.07]
Madagascar	1.40 [0.92, 2.12]	4.76 [1.73, 13.04] *	0.90 [0.61, 1.33]	1.16 [0.69, 1.96]
Malawi	1.24 [0.83, 1.84]	1.29 [0.66, 2.55]	0.81 [0.58, 1.14]	0.49 [0.24, 1.02]
Mali	1.58 [1.03, 2.43] *	2.74 [1.71, 4.39] **	1.15 [0.79, 1.68]	0.92 [0.59, 1.44]
Morocco	7.69 [3.52, 16.81] **	1.74 [0.71, 4.25]	0.79 [0.32, 1.95]	1.66 [0.66, 4.15]
Mozambique	1.40 [0.91, 2.17]	1.51 [0.65, 3.48]	1.02 [0.68, 1.52]	1.45 [0.91, 2.33]
Namibia	1.01 [0.51, 2.03]	1.63 [0.66, 4.01]	1.29 [0.65, 2.51]	1.37 [0.54, 3.52]
Nepal	4.46 [1.61, 12.32] **	1.59 [0.39, 6.42]	0.45 [0.20, 1.04]	1.35 [0.61, 3.00]
Nicaragua	4.13 [2.15, 7.93] **	1.42 [0.64, 3.16]	0.76 [0.39, 1.49]	1.07 [0.56, 2.05]
Niger	1.53 [0.80, 2.91]	1.90 [0.84, 4.30]	0.54 [0.27, 1.09]	1.99 [1.08, 3.67] *
Nigeria	2.14 [1.48, 3.09] **	1.62 [1.10, 2.39] *	0.99 [0.69, 1.41]	0.84 [0.59, 1.19]
Pakistan	3.49 [2.12, 5.73] **	1.07 [0.58, 1.97]	1.03 [0.62, 1.70]	0.74 [0.46, 1.21]
Philippines	5.13 [2.61, 10.08] **	0.62 [0.16, 2.33]	0.41 [0.22, 0.75] **	1.33 [0.73, 2.42]
Rwanda	6.03 [3.61, 10.05] **	1.47 [0.33, 6.59]	1.14 [0.72, 1.79]	1.20 [0.70, 2.05]
Sao Tome & Principe	6.53 [1.02, 41.64] *	1.22 [0.11, 13.30]	2.25 [0.74, 6.85]	1.30 [0.41, 4.08]
Senegal	3.15 [2.03, 4.88] **	1.80 [0.94, 3.46]	1.15 [0.76, 1.73]	1.05 [0.63, 1.74]
Sierra Leone	2.44 [1.16, 5.15] *	1.56 [0.74, 3.32]	1.39 [0.65, 3.02]	0.69 [0.31, 1.52]
Swaziland	2.93 [1.16, 7.37] *	1.57 [0.40, 6.27]	0.91 [0.40, 2.08]	0.38 [0.12, 1.20]
Tanzania	2.75 [1.74, 4.34] **	1.49 [0.38, 5.94]	0.89 [0.60, 1.34]	1.39 [0.76, 2.53]
Timor-Leste	2.13 [1.21, 3.75] **	1.32 [0.84, 2.23]	2.39 [1.26, 4.56] **	1.87 [1.15, 3.05] *
Turkey	6.19 [3.31, 16.55] **	1.88 [0.71, 5.01]	0.62 [0.24, 1.63]	0.96 [0.44, 2.11]
Uganda	1.83 [1.17, 2.86] **	3.68 [1.44, 9.36] **	–	1.23 [0.57, 2.66]
Zambia	1.76 [1.08, 2.86] *	2.52 [1.29, 4.88] **	0.91 [0.60, 1.45]	0.94 [0.54, 1.63]
Zimbabwe	1.47 [0.74, 2.29]	2.25 [1.23, 4.12] **	1.23 [0.66, 2.32]	0.22 [0.07, 0.67] **

** p<0.01,

* p<0.05;

reference category (switching from health facility to home).

The estimated odds ratios and their 95% CIs of switching from home to a health facility from a two-level logistic regression model for the pooled data are presented in Table 4. The country-level variance estimates show significant heterogeneity in switching behaviour between countries after adjusting for relevant predictors. The base model included year of survey and region to account for the period and geographical effects (Model 1). Accounting for the primary factors explained about 14% of the variations in the direction of switching (Model 2). The control variables explained an additional 17% while the contextual variables including interaction effects explained 20% of the variations (Models 3 and 4).

**Table 4:** Odds ratios [95% CI] for switching from home to a health facility: results from two-level random intercept logistic regression models, pooled data.

Background characteristics	Model 1	Model 2	Model 3	Model 4
Year of survey				
2000–2003	1.00	1.00	1.00	
2004–2006	1.02 [0.74, 1.39]	1.02 [0.76, 1.38]	1.03 [0.78, 1.34]	1.02 [0.80, 1.30]
2007–2008	1.15 [0.83, 1.59]	1.11 [0.81, 1.52]	1.20 [0.90, 1.59]	1.17 [0.91, 1.52]
2009–2010	1.09 [0.76, 1.58]	1.06 [0.74, 1.52]	1.13 [0.82, 1.56]	1.13 [0.85, 1.52]
Region				
South & Southeast Asia	1.00	1.00	1.00	
sub-Saharan Africa	0.77 [0.61, 0.97]*	0.66 [0.53, 0.83]**	0.62 [0.51, 0.76]**	0.53 [0.43, 0.65]**
North Africa	0.77 [0.49, 1.21]	0.90 [0.58, 1.39]	0.85 [0.58, 1.26]	1.02 [0.70, 1.47]
Latin America & Caribbean	1.08 [0.76, 1.55]	0.90 [0.64, 1.27]	0.89 [0.65, 1.21]	0.93 [0.70, 1.24]
**Primary variables**				
Parity				
2		1.00	1.00	
3		2.11 [1.92, 2.32]**	2.09 [1.90, 2.30]**	3.34 [2.55, 4.39]**
4+		2.59 [2.39, 2.80]**	2.49 [2.30, 2.70]**	4.83 [3.81, 6.11]**
Antenatal care				
No antenatal visit		1.00	1.00	
1–3 visits		1.43 [1.28, 1.59]**	1.46 [1.31, 1.64]**	1.47 [1.32, 1.64]**
4+ visits		1.89 [1.69, 2.10]**	1.95 [1.75, 2.18]**	1.98 [1.77, 2.21]**
Wealth status				
Poorest		0.91 [0.79, 1.05]	0.80 [0.69, 0.92]**	0.81 [0.70, 0.93]**
Poor		0.90 [0.78, 1.03]	0.80 [0.70, 0.93]**	0.82 [0.71, 0.94]**
Middle		0.90 [0.79, 1.03]	0.83 [0.72, 0.95]**	0.84 [0.73, 0.96]**
Rich		0.9 0[0.79, 1.02]	0.86 [0.75, 0.98]*	0.86 [0.76, 0.98]*
Richest		1.00	1.00	
Place of residence				
Urban		1.00	1.00	
Rural		1.00 [0.92, 1.09]	1.01 [0.92, 1.10]	1.01 [0.93, 1.11]
**Control variables**				
Maternal education				
No formal education				
Primary			0.89 [0.82, 0.98]*	0.90 [0.83, 0.99]**
Secondary or higher			0.76 [0.68, 0.85]**	0.78 [0.70, 0.87]**
Partners educational status				
No formal education			1.00	
Primary			0.93 [0.84, 1.03]	0.94 [0.85, 1.04]
Secondary or higher			0.84 [0.75, 0.93]**	0.84 [0.75, 0.94]**
Missing or don't know			0.94 [0.78, 1.13]	0.96 [0.80, 1.15]
**Contextual factors**				
Infant mortality rate				1.11 [1.07, 1.16]**
**Interaction:**				
parity×Infant mortality rate				
3×Infant mortality rate				0.93 [0.89, 0.97]**
4+× Infant mortality rate				0.90 [0.86, 0.93]**
**Random effect**				
Country level variance	0.07 [0.03, 0.10]**	0.06 [0.03, 0.09]**	0.05 [0.02, 0.07]**	0.03 [0.01, 0.06]**

** *p*<0.01; *p*<0.05; Infant Mortality Rate expressed as infant deaths per 10,000 live births.

**Figure 3:**
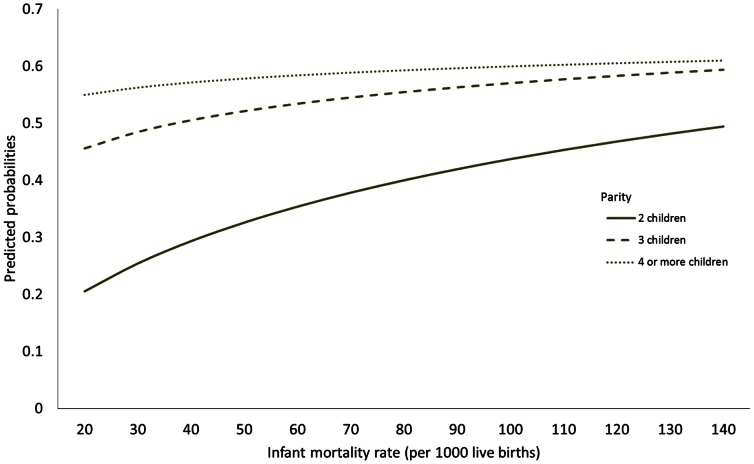
Predicted probability of switching from home to health facility by parity and infant mortality rate.

The year of survey was not significant, confirming that there have been no significant shifts in childbirth from home to health facilities. Regional effects show that women in sub-Saharan Africa are about 47% less likely to switch in favour of a health facility (Model 4) when compared to their counterparts in South and Southeast Asia (*p*<0.01). Considering the primary factors, high parity women are more highly likely to switch from home to a facility when compared to low parity women. However, there exists a significant interaction between parity and infant mortality rates at the country level (Model 4). The direction of switching is in favour of the health facility in countries with high infant mortality rates (*p*<0.01). In these countries, low parity women have an increased probability of switching from home to a health facility (Figure 3).

Women who had antenatal visits are significantly likely to switch their place of childbirth to a facility and the effects are high for women who had four or more antenatal visits (OR: 1.97, 95% CI: 1.77, 2.20, *p*<0.01). Household wealth is significantly associated with switching behaviour; the effects are more pronounced after adjusting for education of mothers and their partners (Models 2 and 3). Poorer women are significantly less likely to switch from home to a health facility. The direction of switching is independent of the place of residence when other variables were included.

Educated women are generally more likely to give birth in a health facility. Nonetheless, those who switch childbirth place favour home over facilities. We tested for potential interaction between education and parity but the results were not significant. The effects were similar for partners' education. Control variables not significant at the 5% level were excluded from the models. None of the contextual variables was statistically significant, except infant mortality rate. The number of years lived in the current place of residence had no significant association with the direction of switching. This suggests that the probability of switching from home to a health facility setting or vice versa is not dependent on migration status.

## Discussion

Our analyses of 44 low and middle income countries demonstrate new evidence that although most women tend to use the same setting (health facility or home) for their successive births, a substantial proportion (about 14%) did switch their childbirth place. Not all these individual decisions favour health facilities. About 50% of women who switch their place of childbirth favour home to a health facility. Nonetheless, there is evidence of behavioural change in the uptake of facility care for childbirth, contributing to the progress towards reduction of maternal mortality [30]–[31]. Our analysis shows that women do not necessarily rely on facilities for their successive parities. Parity, antenatal care use and wealth are strongly associated with the decision to shift towards a home or a facility birth.

The finding that mothers deciding to choose against facility births suggests negative experiences and poor quality maternity care for preceding births. Unfortunately there is no information on the quality of care at birth in the DHS data – so it is not possible to examine this effect. However, the finding that frequent antenatal care visits increase the odds of switching from home to a facility suggests that proper and adequate antenatal care can influence women's decision to move towards the safer childbirth option. Nevertheless, there is also a possible selection effect where mothers with high risk pregnancy seek more antenatal care and may decide to give birth in a facility. This is particularly the case for high parity women. Yet another important result is that switching from a home to a health facility for childbirth or vice versa is independent of migration status, indicating that it is not movement from one place to another that determines the choice of place for childbirth.

With regard to the contextual effects, infant mortality rate has significant impact in determining the choice of place for childbirth care. The significant interaction between infant mortality rate and parity shows that although low parity women have lower probability of switching from home to a health facility, the probability of switching tend to significantly increase for those residing in countries with high infant mortality rate.

Inevitably, the factors associated with health systems play a crucial role in the uptake of facility births [31]–[32]. Poor quality of care can deter women from seeking childbirth care in facilities. The generational change in younger mothers intending to give birth in facilities provide reassurance and hope to reducing maternal mortality rates – a critical goal of the UN Millennium Development Programme. Maternal health interventions should explicitly focus its efforts to promote antenatal and institutional birth care especially in resource poor settings. There is need for further qualitative research to disentangle the socioeconomic and cultural factors influencing women's choices and decision to evade facilities for childbirth care.
